# Pregnancy Vitamin D Deficiency Prevalence and Adverse Birth Outcomes in Mazandaran, a Province of Iran: A Cross‐Sectional Study

**DOI:** 10.1155/jp/3216925

**Published:** 2026-06-29

**Authors:** Mohammad Heidari Seyedmahalleh, Zahra Kashi, Akbar Fazeltabar Malekshah, Mina Khasayesi

**Affiliations:** ^1^ Diabetes Research Center, Imam Teaching Hospital, Mazandaran University of Medical Sciences, Sari, Iran, mazums.ac.ir

**Keywords:** cross-sectional study, fasting blood sugar, maternal health, Mazandaran, neonatal outcomes, pregnancy, public health, seasonal variation, supplementation, vitamin D deficiency

## Abstract

**Background and Objective:**

Vitamin D deficiency (VDD) is a significant health concern among pregnant women, with implications for maternal and neonatal outcomes. This study investigated the prevalence of VDD in Mazandaran and its association with demographic factors, maternal health, and neonatal outcomes.

**Methods:**

A cross‐sectional study was conducted on 233 pregnant women attending prenatal clinics in Mazandaran, Iran, from 2020 to 2023. Participants were recruited using systematic random selection during the first trimester and categorized based on serum Vitamin D levels: severe deficiency (< 10 ng/mL), deficiency (10–20 ng/mL), insufficiency (20–29 ng/mL), and sufficiency (≥ 30 ng/mL). Demographic, biochemical, and pregnancy outcome data were collected. Statistical analyses included ANOVA, chi‐square for comparing means, and scattering assessment.

**Results:**

Mean gestational age was 10.2 weeks. Nearly 73% of women had suboptimal Vitamin D levels, with 24.5% classified as severely deficient. Significant associations were observed between VDD and first‐trimester fasting blood sugar (FBS) measured at the time of blood sampling (*p* = 0.001), and maternal vitamin D supplementation before conception or during early pregnancy was more common among women with sufficient vitamin D (51.1%) than those with deficiency (37.5%) (*p* = 0.033). Neonatal health outcomes, such as hypothyroidism, rheumatic diseases, heart disease, risk of cesarean delivery, and inborn errors of metabolism (IEMs), were not significantly associated with maternal VDD (*p* = 0.208). Although specific complications, such as low birth weight, were not statistically significant (*p* = 0.381).

**Conclusion:**

The high prevalence of VDD among pregnant women in Mazandaran, coupled with its association with maternal and neonatal health factors like maternal blood glucose level and supplementation before and during pregnancy, highlights the need for targeted interventions.

## 1. Introduction

Vitamin D is a fat‐soluble vitamin that is essential for maintaining calcium and phosphorus homeostasis, critical for fetal skeletal development, and maternal bone health [1]. Vitamin D also plays a pivotal role in immune function and cell differentiation [2, 3]. Vitamin D deficiency (VDD) refers to suboptimal serum levels of 25‐hydroxyvitamin D [25(OH)D] and according to the Institute of Medicine (IOM), VDD is classified as severe when serum 25(OH) D levels are below 10 ng/mL, deficient between 10 and 20 ng/mL, insufficient between 20 and 29 ng/mL, and sufficient when ≥ 30 ng/mL [4]. VDD is a major global health concern, particularly among pregnant women [5, 6]. Its deficiency during pregnancy has been associated with serious complications, including preeclampsia, gestational diabetes, preterm labor, and increased risk of infections in the mother [7–9]. For neonates, maternal VDD can lead to low birth weight (LBW), intrauterine growth restriction (IUGR), and neonatal hypocalcemia [10]. Emerging evidence also suggests that inadequate maternal vitamin D may have long‐term effects on the child′s immune function and risk of chronic diseases such as asthma and Type 1 diabetes [11].

Globally, the prevalence of VDD among pregnant women varies widely, ranging from 20% in developed countries to as high as 85% in developing nations [12]. In the Middle East and South Asia, factors such as limited sun exposure due to cultural clothing practices, air pollution, and inadequate dietary intake of vitamin D exacerbate this issue [13]. Several studies have highlighted the widespread nature of VDD among pregnant women from different parts of Iran with various cultural and regional characteristics [14–17]. Reports from various regions in Iran indicate a high prevalence of VDD among pregnant women, with estimates ranging from 60% in Tehran to over 80% in regions such as Shahroud, Zanjan, and Isfahan [15, 18, 19]. These findings highlight significant regional variability, likely influenced by geographic, seasonal, and lifestyle factors [18].

Despite these alarming statistics, evidence from northern Iran, particularly the Mazandaran province, is scarce. Cultural practices, including clothing habits and time spent indoors, might further contribute to VDD in this region [20]. To date, no comprehensive studies have assessed the prevalence of VDD among pregnant women in Mazandaran, representing a critical knowledge gap. This study is aimed at addressing this gap by estimating the prevalence of VDD among pregnant women in Mazandaran and examining its association with demographic, anthropometric, and biochemical factors. Additionally, this research seeks to identify potential correlations between maternal VDD and adverse pregnancy outcomes.

## 2. Method

### 2.1. Study Design and Participants

**Figure 1:**
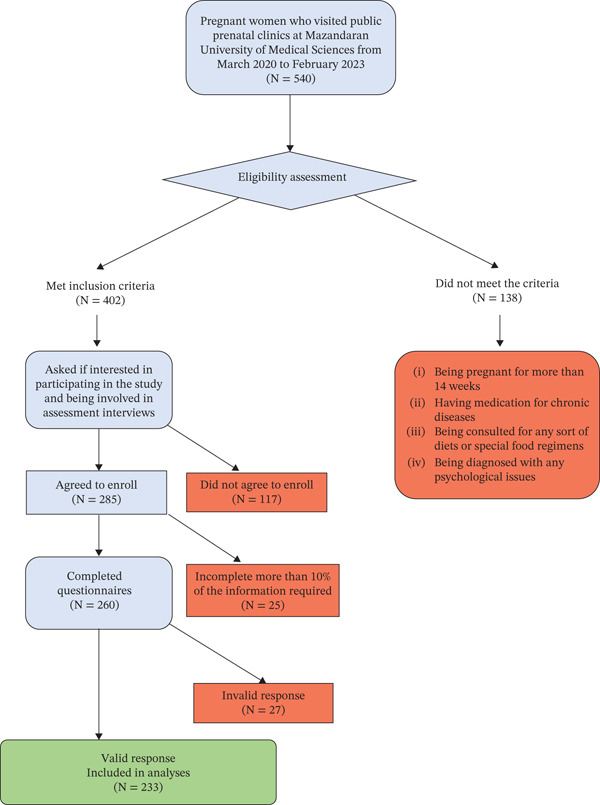
Participant selection flow chart diagram.

This cross‐sectional study was conducted from March 2020 to February 2023 in Mazandaran province, northern Iran. Participants were recruited from public prenatal clinics affiliated with Mazandaran University of Medical Sciences, located primarily in the city of Sari. A total of 233 pregnant women in their first trimester (≤ 14 weeks′ gestation); gestational age was obtained from last menstrual period (LMP) and confirmed by 1st‐trimester ultrasound when available, were enrolled using systematic random sampling. Eligibility criteria included being under 14 weeks pregnant, permanent residency in Mazandaran for at least 6 months, and willingness to provide informed consent. Exclusion criteria included a medication for chronic diseases (renal, hepatic, or thyroid disorders), conditions affecting calcium or vitamin D metabolism, and the use of high‐dose vitamin D supplements within the previous 3 months, as presented according to the CONSORT flow diagram (Figure 1). Preterm births and LBW outcomes were not excluded a priori and were captured from delivery records to allow descriptive analysis. This study was approved by the Ethics Committee of Mazandaran University of Medical Sciences (Approval ID: IR.MAZUMS.REC.1398.1208). Written informed consent was obtained from all participants before enrollment. Participants identified with severe VDD (serum 25[OH] D <10 ng/mL) were provided counseling and referred to a clinical nutritionist for further evaluation and appropriate supplementation, following ethical clinical care protocols. Confidentiality and anonymity of participant data were strictly maintained throughout the study.

### 2.2. Sampling Method

Systematic random sampling was employed to recruit participants from eligible prenatal clinics. Participants were prospectively enrolled by systematic random sampling at first‐trimester clinic visits; pregnancy and neonatal outcomes were later abstracted from routine antenatal and delivery records and linked to the baseline record. Thus, sampling was prospective for exposure ascertainment, with prospective follow‐up through record abstraction for delivery outcomes. The required sample size was calculated based on an expected prevalence of VDD of 70%, derived from prior literature reporting similar rates among pregnant women in Iran [15]. The sample size was estimated with a 95% confidence level (*Z* = 1.96) and a 6% margin of error, using the standard formula [21]:

n=Z2×p1−pd2

This calculation yielded a minimum required sample size of 225 participants.

### 2.3. Assessment and Measurements

#### 2.3.1. Demographic and Anthropometric Data

Data on age, education level, socioeconomic status, occupation, clothing habits, and sun exposure were collected via a structured questionnaire previously validated and applied in Iranian surveillance of risk factors of noncommunicable diseases (ISRFNCD) [22]. This questionnaire captures the data regarding the age, gender, education level, living condition, employment, marital status, disease duration, mode of treatment, and related comorbidity factors of the patients. Body weight and height were measured to calculate BMI using the formula pre‐pregnancy weight (kg)/height (m^2^). Pre‐pregnancy BMI (kg/m^2^) was abstracted from antenatal records when documented; otherwise, first‐visit measured weight and recorded height were used as a proxy. Participants were categorized into underweight (< 18.5 kg/m^2^), normal (18.5–24.9 kg/m^2^), overweight (25–29.9 kg/m^2^), and obese (≥ 30 kg/m^2^).

#### 2.3.2. Biochemical Analysis

Fasting blood samples (5 mL) were collected from each participant at the first‐trimester (≤ 12 weeks) visit concurrent with vitamin D sampling. Serum levels of 25(OH)D, calcium, and phosphorus were assessed through a validated chemiluminescent immunoassay (CLIA) method. The 25(OH)D and biochemical markers were measured using high‐sensitivity diagnostic kits manufactured by DiaSorin (Italy), and testing was conducted at a single centralized laboratory under controlled conditions to ensure consistency. Blood samples were centrifuged, and serum was stored at −80°C until analysis. The DiaSorin LIAISON XL system used for the analysis had a coefficient of variation (CV) of less than 8%, and its lower detection limit for 25(OH)D was 4 ng/mL, ensuring accurate and reproducible measurements. The analyses were performed using the DiaSorin LIAISON XL analyzer, with a 4 ng/mL sensitivity threshold. Laboratory tests were conducted at the Central Clinical Laboratory of Imam Khomeini Teaching Hospital in Sari. Vitamin D status was categorized as: Severe deficiency (< 10 ng/mL), deficiency (10–20 ng/mL), insufficiency (21–29 ng/mL), and sufficiency (≥30 ng/mL) according to the IOM standards and preliminary literature [4, 23, 24].

#### 2.3.3. Pregnancy Outcomes

Pregnancy‐related outcomes, including preeclampsia, gestational diabetes, and IUGR, were extracted from medical records and confirmed based on clinical diagnostic criteria documented by attending obstetricians. Neonatal outcomes such as birth weight, length, and head circumference were measured immediately after birth in the delivery or reception room using calibrated hospital‐grade instruments. Birth weight was recorded with a digital infant scale (Seca, Germany) with minimal clothing, and length was measured using an infantometer. Head circumference was assessed using a nonstretchable measuring tape from the most prominent part of the occiput to the mid‐forehead. Apgar scores at 1 and 5 min were recorded by trained staff according to standard clinical practice, with low scores (< 7) flagged for potential birth asphyxia. LBW < 2500 g; preterm birth < 37 completed weeks (from LMP or 1st‐trimester ultrasound); small‐for‐gestational‐age (SGA) and large‐for‐gestational‐age (LGA) were defined using local reference percentiles by sex and gestational age, based on LMP or first‐trimester ultrasound when LMP was uncertain. Data regarding mothers′ supplemental use, types, doses, and frequencies were collected; however, due to inconsistencies in recording or uncertainty of reporting and recording of the data since it was not under the supervision of well‐trained staff, we preferred not to extract or apply these data from their antenatal files. All neonatal complications were reviewed and confirmed by a neonatologist. The data were analyzed using chi‐square tests for categorical variables and analysis of covariance (ANCOVA) for continuous neonatal outcomes, adjusting for gestational age and infant sex.

### 2.4. Statistical Analysis

Data were analyzed using SPSS software (Version 22). Statistical tests are included as follows: Descriptive statistics are reported as means and standard deviations for continuous variables, and frequencies and percentages for categorical variables. We implemented group comparison analysis through independent *t*‐tests and ANOVA for continuous variables and chi‐square tests for categorical data. Also, we considered confounding variables via multivariate logistic and linear regression analysis, including mothers′ education, mothers′ job, familial income, history of VDD, history of GDM, DM, or HTN, and supplemental use before pregnancy. A *p* value < 0.05 was considered statistically significant.

## 3. Results

### 3.1. Baseline Characteristics of Participants

Table 1 presents the baseline characteristics of the 233 pregnant women included in the study. Most participants (50.2%) were aged 30–43 years. Most women had a pre‐pregnancy BMI in the obese category (40.8%), followed by those with a normal BMI (28.8%). Vitamin D supplementation before pregnancy was reported by 43% of women, whereas 57% did not take supplements. A significant proportion of participants (88.5%) were not employed. Regarding education level, 89% of the women had only completed school, whereas a smaller percentage held a bachelor′s (8.7%) or master′s degree (2.3%). Blood samples were predominantly collected during the year′s second half (82.9%).

**Table 1:** Baseline characteristics of 233 pregnant women.

Variables		Count *N*(%)
Vitamin D status	SVDD (< 10 ng/mL)	57 (24.5)
	VDD (10–20 ng/mL)	77 (33)
	VDIS (20–29 ng/mL)	36 (15.5)
	VDS (≥ 30 ng/mL)	63 (27)
Age	16–29 (years)	116 (49.8)
	30–43 (years)	117 (50.2)
BMI	Underweight (< 18.5 kg/m^2^)	6 (2.6)
	Normal (18.5–24.9 kg/m^2^)	67 (28.8)
	Overweight (25–29.9 kg/m^2^)	65 (27.9)
	Obese (≥ 30 kg/m^2^)	95 (40.8)
FBS	Normal (< 92 mg/dL)	51 (68.9)
	High (≥ 92 mg/dL)	23 (31.1)
Season^a^	First half	40 (17.1)
	Second half	193 (82.9)
Supplementation^b^	Yes	100 (43)
	No	133 (57)
Mother′s education	School graduated	154 (89)
	Bachelor	15 (8.7)
	Masters	4 (2.3)
Mother′s occupation	Occupied	22 (11.5)
	Not occupied	167 (88.5)
Familial income	Lowest (1st tertile)	86 (36.9)
	Moderate (2nd tertile)	73 (31.3)
	Highest (3rd tertile)	74 (31.8)

Abbreviations: BMI, body mass index; FBS, fasting blood sugar; SVDD, severe vitamin D deficiency; VDD, vitamin D deficiency; VDIS, vitamin D insufficiency; VDS, vitamin D sufficiency.

^a^Season of collecting blood samples.

^b^Vitamin D supplementation before pregnancy.

### 3.2. Vitamin D Status and Participant Characteristics

Table 2 shows the distribution of participant characteristics across the four Vitamin D status groups. The mean serum Vitamin D level for the total population was 21.6 ng/mL (SD = 16), with a significant variation across groups (*p* < 0.001). The VDS group had the highest mean level at 46.2 ± 12.9 ng/mL, whereas the SVDD group showed the lowest at 6.7 ± 1.8 ng/mL (Table 2). Fasting blood sugar (FBS) levels significantly differed by vitamin D status (*p* = 0.001), with the highest FBS in the VDD group (89.8 ± 12.7 mg/dL) and the lowest in the VDS group (78.8 ± 8.4 mg/dL). Although the distribution of samples by season showed higher VDD in the first half of the year (20%), this difference was not statistically significant (*p* = 0.192). Vitamin D supplementation before pregnancy was reported by 37.5% of the VDD group and 51.1% of the VDS group (*p* = 0.033). Educational level and maternal occupation did not differ significantly across groups.

**Table 2:** Conditions and characteristics of participants in total and Vitamin D status groups.

Variables		Total^a^	VDD^a^	VDS^a^	*p* ^b^
Vitamin D status (ng/mL)		21.6 (16)	14.2 (6.9)	46.2 (12.9)	**< 0.001**
Age (years)		28.8 (6.2)	29.1 (5.9)	28 (6.9)	0.266
BMI (kg/m^2^)		26.9 (5.5)	26.9 (5.4)	26.7 (5.8)	0.876
FBS (mg/dL)		87.3 (12.7)	89.8 (12.7)	78.8 (8.4)	**0.001**
Season^c^	First half	15 (16.8)	13 (20)	2 (8.3)	0.192
	Second half	74 (83.2)	52 (80)	22 (91.7)	
Supplementation^d^	Yes	78 (37.5)	55 (33.7)	23 (51.1)	**0.033**
	No	130 (62.5)	108 (66.3)	22 (48.9)	
Mother′s Education	School graduated	154 (89)	115 (87.8)	39 (74.6)	0.791
	Bachelor	15 (8.7)	13 (9.9)	2 (4.8)	
	Masters	4 (2.3)	3 (2.3)	1 (2.4)	
Mother′s occupation	Occupied	24 (12.5)	21 (14.4)	3 (6.7)	0.358
	Not occupied	167 (87.5)	125 (85.6)	42 (93.3)	
Familial income	Lowest (1st tertile)	83 (36.9)	54 (62.8)	32 (37.2)	0.321
	Moderate (2nd tertile)	73 (31.3)	38 (52.1)	35 (31.3)	
	Highest (3rd tertile)	74 (31.8)	46 (62.2)	28 (37.8)	
Mother′s number of pregnancies	One	67 (31.9)	49 (30.4)	18 (36.7)	0.686
	Two	97 (46.2)	78 (48.4)	19 (38.8)	
	Three	28 (13.3)	20 (12.4)	8 (16.3)	
	Four to six	18 (8.6)	14 (8.6)	4 (8.1)	
Parity	Nulliparous	229 (98.3)	139 (98.5)	90 (97.7)	0.647
	Multiparous	4 (1.7)	2 (1.5)	2 (2.3)	
History of VDD	Yes	74 (34.7)	57 (33.9)	17 (37.8)	0.630
	No	139 (65.3)	111 (66.9)	28 (62.2)	
History of GDM	Yes	11 (5.2)	10 (6.1)	1 (2.2)	0.714
	No	200 (94.8)	155 (93.9)	45 (97.8)	
History of DM	Yes	14 (6.6)	11 (6.6)	3 (6.5)	0.987
	No	199 (93.4)	156 (93.4)	43 (93.5)	
History of HTN	Yes	5 (2.4)	3 (1.8)	2 (4.4)	0.313
	No	203 (97.6)	160 (98.2)	43 (95.6)	

*Note:* Statistically significant values (*p* < 0.05) are bolded.

Abbreviations: BMI, body mass index; DM, diabetes mellitus; FBS, fasting blood sugar; GDM, gestational diabetes mellitus; HTN, hypertension; VDD, vitamin deficiency; VDS, vitamin D sufficiency.

^a^All values are means (standard deviation [SD]) for continuous variables or number (percent) for categorical variables.

^b^Obtained from ANOVA for continuous variables and chi‐square test for categorical variables.

^c^Season of first‐trimester blood sampling (≤ 14 weeks): first half (spring–summer) versus second half (autumn–winter).

^d^Vitamin D supplementation before pregnancy.

### 3.3. Newborn Conditions and Delivery Outcomes

Table 3 summarizes the characteristics of newborns and delivery outcomes across Vitamin D status groups. The mean birth weight for the total population was 3177.1 g (SD = 568.7), with no significant differences across groups (*p* = 0.104). No significant association was found between vitamin D status and newborn health condition (*p* = 0.208). Cesarean section (CS) was the most common delivery method (69.1%). Although this was statistically insignificant between different delivery types (*p* = 0.704), the majority of the CS deliveries were assigned to the VDD group (80%). Results were insignificant regarding the weight for length of the neonates (*p* = 0.381).

**Table 3:** Newborn conditions and delivery outcomes of pregnant women in total and Vitamin D status groups.

Variables		Total	VDD^a^	VDS^a^	*p* ^b^
Birth weight (g)		3177.1 (568.7)	3137.1 (577.5)	3322 (518.5)	0.104
Birth height (Cm)		50.4 (3.2)	50.4 (3.4)	50.4 (2.7)	0.968
Delivery type	CS	103 (69.1)	80 (68.4)	23 (71.9)	0.704
	NVD	46 (30.9)	37 (31.6)	9 (28.1)	
Sex	Boy	78 (52.7)	63 (54.3)	15 (46.9)	0.456
	Girl	70 (47.3)	53 (45.7)	17 (53.1)	
Newborn′s health condition	Normal	123 (52.8)	94 (55.3)	29 (46)	0.208
	With conditions	110 (47.2)	76 (44.7)	34 (54)	
Weight for gestational age	SGA (< 5%)	14 (9.5)	13 (11.2)	7 (28)	0.381
	LGA (> 95%)	8 (5.4)	6 (5.2)	2 (6.3)	
Birth weight^c^	NBW	196 (84.1)	118 (85.5)	78 (82.1)	0.750
	LBW	23 (9.9)	12 (8.7)	11 (11.6)	

Abbreviations: CS, cesarean section; LBW, low birth weight; LGA, large for gestational age; NBW, normal birth weight; NVD, natural vaginal delivery; SGA, short for gestational age; VDD, vitamin D deficiency; VDS, vitamin D sufficiency.

^a^All values are means (standard deviation [SD]) for continuous variables or number (percent) for categorical variables.

^b^Obtained from ANOVA for continuous variables and chi‐square test for categorical variables.

^c^Birth weights in the range of 2500–4500 g were considered normal, and below it, as low birth weight.

## 4. Discussion

This study is aimed at investigating the prevalence of VDD among pregnant women in Mazandaran and assess its association with demographic factors, maternal health, and neonatal outcomes. The findings revealed that nearly 73% of participants had suboptimal vitamin D levels, with 24.5% classified as severely deficient. Significant associations were observed between vitamin D status and FBS and maternal supplementation. These results underscore the public health importance of vitamin D in maternal and neonatal health, particularly in regions with high VDD prevalence like northern Iran [25, 26].

The prevalence of VDD in our study aligns with global and national trends but demonstrates some regional disparities. For example, a study in Tehran reported a VDD prevalence of 81%, similar to the 78% found in Yazd and 86% in Zanjan, particularly during winter [15–17]. In Mazandaran, the overall prevalence was slightly lower, potentially due to differences in seasonal sunlight exposure, dietary patterns, and cultural habits [27]. A cross‐sectional study carried out on the general population of Sari (the capital city of Mazandaran Province) in 2011 revealed a significant seasonal variation, with deficiency rates of 78.6% in summer and 87.5% in winter, highlighting the impact of limited sun exposure and lifestyle factors [28]. The significant seasonal variation in vitamin D levels observed in this study, with higher deficiencies during the first half of the year, is consistent with findings from Zanjan and Yazd, where limited sun exposure during colder months and cultural clothing practices contribute to VDD [15, 16]. However, the slightly lower prevalence in Mazandaran may reflect regional dietary differences, such as the consumption of vitamin D‐rich fish, a more common practice in coastal areas [29, 30]. Nevertheless, the high prevalence across all seasons highlights persistent challenges such as inadequate dietary supplementation and limited public awareness about sun exposure and vitamin D.

Vitamin D plays a critical biological role in calcium and phosphorus homeostasis, essential for fetal bone development, maternal metabolic health, and immune regulation [3, 31, 32]. Vitamin D regulates cell proliferation, differentiation, and immune responses by modulating cytokine secretion, reducing NF‐*κ*B expression, and preventing inflammation in myometrial cells through the nuclear factor kappa B pathway [33]. The association between severe VDD and elevated FBS in this study aligns with evidence that vitamin D modulates insulin secretion and sensitivity through its receptors on pancreatic *β*‐cells, activation of the insulin receptor gene, and modulation of insulin‐mediated glucose transport, supported by its enzymatic and genetic interactions in *β*‐cells and skeletal muscle [34, 35]. Impaired glucose metabolism in vitamin D‐deficient individuals increases the risk of gestational diabetes, potentially complicating pregnancy outcomes [36]. Moreover, maternal vitamin D levels are directly related to placental health and calcium transfer [37], which are critical for optimal fetal growth and development [32, 38]. Although significant associations between maternal vitamin D levels and neonatal birth weight or delivery type were not observed in this study, previous research suggests that severe maternal VDD may increase the risk of LBW, preterm delivery, and neonatal hypocalcemia [39, 40]. The lack of statistical significance in our findings could be attributed to the sample size or other confounding factors, but the trends observed warrant further investigation into the impact of maternal vitamin D status on neonatal health outcomes.

Demographic factors, including maternal education, were significantly associated with vitamin D status. Women with higher education levels were more likely to have sufficient vitamin D levels, possibly due to greater awareness and adherence to nutritional guidelines [41]. However, no significant differences were observed for maternal age or BMI, suggesting that lifestyle and environmental factors may play a more prominent role in influencing vitamin D levels during pregnancy in this population [42].

Findings from the association analysis revealed no significant links between maternal and neonatal characteristics and mothers′ serum level of vitamin D. These relationships remained insignificant after adjusting for confounding variables like: Mothers′ education, mothers′ job, familial income, history of VDD, history of GDM, DM, or HTN, supplemental use before pregnancy (Table S1).

The strengths of this study include its comprehensive approach, which combined biochemical assessments with demographic and clinical data to provide a detailed understanding of VDD in pregnant women. The study also addresses a critical knowledge gap in northern Iran, contributing valuable data for public health planning. However, the study has several limitations. Its cross‐sectional design precludes establishing causal relationships between vitamin D levels and health outcomes. Even though VD food sources are scarce in Iranian traditional food choices, not employing them in our study would be considered a limitation. Moreover, the lack of time and area of skin exposed to sunlight data, mostly due to religious considerations, would be one of our most critical limitations. Also, the small number of the target population and the statistical power of the findings could be other limitations of our work. Although the sample size used was calculated to have the necessary statistical adequacy, conducting population and prospective studies could strengthen the evidence needed to conclude. The study′s focus on one clinical center′s visitors may also reduce its generalizability to all pregnant women in Mazandaran.

Future research should adopt longitudinal designs to better elucidate causal relationships between maternal vitamin D status and pregnancy outcomes. Studies incorporating detailed dietary assessments, genetic predispositions, and seasonal exposure variations are also needed. Moreover, interventional trials evaluating the impact of vitamin D supplementation on maternal and neonatal health outcomes could inform more effective public health strategies.

## 5. Conclusion

This study highlights the high prevalence of VDD among pregnant women in Mazandaran and its association with adverse maternal and neonatal health conditions. Although significant relationships with specific neonatal complications were not observed, the findings emphasize the importance of adequate vitamin D levels for maternal and fetal health. Targeted interventions, including education, supplementation programs, and awareness campaigns, are crucial to address this public health issue and improve pregnancy outcomes in northern Iran.

## Author Contributions

A.F.M. and Z.K. designed the study, and M.K. carried it out. M.H.S. analyzed the data and interpreted the findings. M.H.S. and Z.K. drafted the manuscript, and A.F.M. and M.K. revised the final manuscript. M.K. commented on the presentation of data, and her comments improved the quality of the paper significantly.

## Funding

This study was supported by Mazandaran University of Medical Sciences (10.13039/501100004160).

## Ethics Statement

All methods followed the relevant guidelines and regulations for studies involving human participants and were reviewed and approved by the Human Ethical Committee of Mazandaran University of Medical Sciences (IR.MAZUMS.REC.1398.1208).

## Consent

The patients/participants provided their written informed consent to participate in this study.

## Conflicts of Interest

The authors declare no conflicts of interest.

## Supporting information


**Supporting Information** Additional supporting information can be found online in the Supporting Information section. Table S1: Logistic and linear regression analysis results representing the associations between anthropometric and biochemical characteristics of 233 pregnant women.

## Data Availability

The authors elect not to share the data sets used and/or analyzed.
