# Supplementary material for: Pregnancy Vitamin D Deficiency Prevalence and Adverse Birth Outcomes in Mazandaran, a Province of Iran: A Cross‐Sectional Study
Source: J Pregnancy. 2026 Jun 29;2026:3216925. doi: 10.1155/jp/3216925 (PMC13314719; doi:10.1155/jp/3216925)
Supplement: Supplementary file 1 — Supporting Information Additional supporting information can be found online in the Supporting Information section. Table S1: Logistic and linear regression analysis results representing the associations between anthropometric and biochemical characteristics of 233 pregnant women. [file JP-2026-3216925-s001.docx]

**Supplementary Table 1.** Associations between anthropometric and biochemical characteristics of 233 pregnant women

| **Variable** | **model** | **β (P-value) Linear Regression Models** | **OR (p-value) Logistic Regression Models** | |
| --- | --- | --- | --- | --- |
|  |  |  | **VDD** | **VDS** |
| BMI (Overweight, Obese Vs Normal) | Crude ^a^ | 0.024 (0.712) | 0.992 (0.978) | 1 (Reference Category) |
|  | Adjusted ^b^ | 0.020 (0.767) | 1.14 (0.963) | 1 (Reference Category) |
| FBS (Normal Vs High) | Crude ^a^ | -0.076 (0.248) | 1.715 (0.202) | 1 (Reference Category) |
|  | Adjusted ^b^ | -0.071 (0.288) | 1.819 (0.176) | 1 (Reference Category) |
| Birth Condition (With Vs without) | Crude ^a^ | N/A | 1.02 (0.941) | 1 (Reference Category) |
|  | Adjusted ^b^ |  | 1 (0.986) | 1 (Reference Category) |
| BW (low Vs. High) | Crude ^a^ | N/A | 0.748 (0.435) | 1 (Reference Category) |
|  | Adjusted ^b^ |  | 0.778 (0.486) | 1 (Reference Category) |
| Abbreviations: VDD: Vitamin D Deficiency; VDS: Vitamin D Sufficiency; BMI: Body Mass Index; FBS: Fasting Blood Sugar; BW: Birth Weight; N/A: Not Applicable; OR: Odds Ratio  ^a^ not adjusted for any confounding variable  ^b^ Adjusted for confounding variables: Mothers’ Education, Mothers ' Job, Familial Income, History of VDD, History of GDM, DM, or HTN, Supplemental use before pregnancy, as a multivariate logistic regression or multivariate linear regression | | | | |
